# Antioxidant Effect of Cocoa By-Product and Cherry Polyphenol Extracts: A Comparative Study

**DOI:** 10.3390/antiox9020132

**Published:** 2020-02-03

**Authors:** Francesca Felice, Angela Fabiano, Marinella De Leo, Anna Maria Piras, Denise Beconcini, Maria Michela Cesare, Alessandra Braca, Ylenia Zambito, Rossella Di Stefano

**Affiliations:** 1 Cardiovascular Research Laboratory, Department of Surgical, Medical and Molecular Pathology and Critical Care Medicine, University of Pisa, 56100 Pisa, Italy; maria.cesare@student.unisi.it (M.M.C.); rossella.distefano@unipi.it (R.D.S.); 2 Department of Pharmacy, University of Pisa, 56126 Pisa, Italy; angela.fabiano@unipi.it (A.F.); marinella.deleo@unipi.it (M.D.L.); anna.piras@unipi.it (A.M.P.); denisebeconcini@gmail.com (D.B.); alessandra.braca@unipi.it (A.B.); ylenia.zambito@unipi.it (Y.Z.); 3 Interdepartmental Research Center “Nutraceuticals and Food for Health”, University of Pisa, 56100 Pisa, Italy; 4 Department of Life Sciences, University of Siena, Siena 53100, Italy

**Keywords:** cocoa by-products, cherry extract, oxidative stress, human endothelial cell

## Abstract

Background: Recent studies have highlighted the importance of cherry and cocoa extracts consumption to protect cells from oxidative stress, paying particular attention to cocoa by-products. This study aims to investigate the protective effect of cocoa husk extract (CHE) and cherry extracts (CE) against ROS-induced oxidative stress in Human Umbilical Vein Endothelial Cells (HUVECs). Methods: CE and CHE had antioxidant activity characterized by total polyphenols content (TPC). HUVECs were treated for 2 h and 24 h with increasing TPC concentrations of CE and CHE (5-10-25-50-100 µg Gallic Acid Equivalent (GAE)/mL) and then with H_2_O_2_ for 1 h. Cell viability and ROS production were evaluated. CE and CHE polyphenols permeability on excised rat intestine were also studied. Results: CE and CHE showed a similar antioxidant activity (2.5 ± 0.01 mmol Fe^2+^/100 g FW (fresh weight) and 2.19 ± 0.09 mmol Fe^2+^/100 g FW, respectively, *p* > 0.05) whereas CHE had a higher TPC (7105.0 ± 96.9 mg GAE/100 g FW) than CE (402.5 ± 8.4 mg GAE/100 g), *p* < 0.05. The in vitro viability assay showed that both extracts were non-cytotoxic. CHE resulted in protection against ROS at lower concentrations than CE. CHE showed a 2-fold higher apparent permeability compared to CE. Conclusions: CHE represents a high-value antioxidant source, which is interesting for the food and pharmaceutical industries.

## 1. Introduction

Reactive oxygen species (ROS) are involved in the pathogenesis of numerous chronic and degenerative diseases. Physiologically, oxygen metabolism generates ROS, which are contrasted and neutralized by antioxidant defenses. The unbalance toward ROS formation is recognized as a critical aspect of cell damage that characterizes many disease states, such as atherosclerosis and premature aging [1,2,3].

Vascular endothelial cell lines are particularly sensitive to ROS, and damage to them is reflected in the alteration of vascular tone and permeability, and thus involved in cardiovascular dysfunction associated with hypertension, diabetes, atherosclerosis, and ischemic heart disease [4,5,6].

Therefore, the use of nutraceuticals appears essential for the prevention and control of ROS induced damages. In nature, polyphenols are the most abundant category of antioxidants. Most of them are in fruits and vegetables and are typically associated with healthy diets. Actually, epidemiological studies and associated meta-analyses have confirmed that their long-term consumption correlates with protection against the development of serious diseases such as cancers, cardiovascular diseases (CVD), diabetes, osteoporosis, and neurodegenerative pathologies [7].

Cocoa and its derivative products are rich in polyphenols, which possess an antioxidant capacity and are associated with the prevention of diseases related to oxidative stress. Most of the phenolic compounds found in cocoa are represented by flavonols, such as catechins, or flavan-3-ols (37%), anthocyanins (4%), and proanthocyanidins (58%) [8,9]. The protective activity of cocoa seems to be due to its phytochemical constituents, especially catechins.

Cocoa by-products, mainly cocoa husk, are produced worldwide in large amounts, constituting about 75% wt of whole fruit from the cocoa harvest [10]. Being a production waste, they are generally discarded, with a negative environmental impact [11]. Moreover, the cocoa by-products (bean and husks) possess nutritional and functional properties, particularly related to the presence of proanthocyanidins identified in the husks [12]. The proanthocyanidins found in husk are tannins, which can have different molecular weights, according to the degree of polymerization [13]. In vitro cellular studies have also confirmed that proanthocyanidins possess antioxidant activity [8,14]. Additionally, flavanols protect cells from oxidative stress by reducing ROS production and inhibiting the activation of caspase-3. Procyanidin B2 also increases the performance of enzymes specifically involved in antioxidant and detoxification processes [15].

Along with cocoa, cherry fruits have valuable nutritional properties, and their beneficial effects have been demonstrated against oxidative stress damage on both neuronal and intestinal epithelial cells. Similarly to cocoa, the most representative nutraceutical actives in cherries are polyphenols, including flavonoids and anthocyanins [16]. Many studies have used natural extracts on endothelial progenitor cells [17,18] or Human Umbilical Vein Endothelial Cells (HUVECs) [19,20,21] in in vitro experiments related to vascular dysfunction.

The aim of the present research was to compare the antioxidant properties of two antioxidant-rich natural products plant extracts, namely cherry and cocoa (bean and husk). The extracts were evaluated both in vitro and ex vivo in order to compare their beneficial effects on vascular related dysfunction upon oral intake.

For this purpose, we performed phenol cocoa bean and husk extraction from two different plant varieties (Costa Rica and Madagascar) that had their antioxidant activity, total polyphenols content, and phenolic composition characterized by HPLC coupled to electrospray ionization tandem mass spectrometry (ESI-MS/MS). The characterization of cherry extract (CE) has already been reported [22]. Moreover, Costa Rica cocoa husk extract (CHE) and CE were compared for their ability to permeate across excised rat intestine.

## 2. Materials and Methods

### 2.1. Materials

Hexane, acetone, and Folin-Ciocalteau reagent, and gelatin were purchased from Sigma-Aldrich (Milan, Italy). HPLC grade formic acid and methanol were purchased from VWR (Milan, Italy). HPLC grade water (18 mΩ) was obtained by a Mill-Q purification system (Millipore Corp., Bedford, MA, USA).

H_2_O_2_ was purchased from Farmac-Zabban S.p.a. (Calderara di Reno, BO, Italy). Medium 199 (M199), fetal bovine serum (FBS), penicillin-streptomycin solution, L-glutamine, and HEPES buffer were supplied by Hospira S.r.l. (Naples, Italy).

4-[3-(4-iodophenyl)-2-(4-nitrophenyl)-2H-5-tetrazolium]-1,3-benzenedisulfonate (WST-1 assay), was purchased from Roche Applied Science (Mannheim, Germany), 5-(and-6)-chloromethyl-2′, 7′-dichloro-di-hydro-fluorescein diacetate, and acetyl ester (CM − -H_2_DCFDA) were supplied by Thermo Fisher Scientific Inc. (Waltham, MA, USA).

### 2.2. Sample Preparation

#### 2.2.1. Cocoa Bean and Husk Phenol Extraction

The cocoa bean and husk phenol extraction were carried out using a procedure reported by Hammerstone et al. [23], slightly modified. Two varieties of cocoa (Costa Rica and Madagascar) were ground for 30 s in order to obtain a homogeneous material that was defatted four times with 125 mL of hexane for 20 min at 200 rpm and subsequently centrifuged for 30 min at 4000 rpm. The defatted cocoa sample was then extracted four times with acetone 70% *v/v* at a ratio of 1:5, stirred for 3 min, and centrifuged for 30 min at 4000 rpm. The extraction procedure was repeated four times after which the supernatants were combined, filtered with a paper filter and the organic solvent was removed by evaporation at room temperature for 48 h. To obtain a stable product the remaining water suspension was lyophilized.

The cocoa from Madagascar was selected because Bruna et al. [24], comparing the content of polyphenols in cocoa husks from different countries (Ghana, Madagascar, Ecuador, Trinidad, and Venezuela), found that Madagascar husks were the richest. The cocoa from Costa Rica was chosen because compared to that from other countries (Ivory Coast, Venezuela, Samoa, Trinidad, Brazil, Ghana, Ecuador, Jamaica), it was found to be particularly rich in epicatechin [25,26,27,28].

#### 2.2.2. Cherry Extract (CE) Preparation and Characterization

The cherry extracts were obtained from the Crognola Capannile variety of *Prunus avium* L., an ancient Tuscan variety of sweet cherry, as described by Beconcini et al. [29].

The Crognola Capannile variety was used for the present study due to their high polyphenol content, as reported by Berni et al. [22].

### 2.3. HPLC-PDA/UVvis-ESI-MS/MS Analysis of Cocoa Extracts

Cocoa extracts were dissolved in methanol at a final concentration of 2.5 mg/mL, then centrifuged for 5 min at 1145× *g*. The supernatants (20 μL injection volume) were subjected to HPLC coupled with a photodiode array (PDA)/UVvis and an ion trap ESI-MS. The LC system was composed of a Surveyor autosampler, a Surveyor LC pump, a Surveyor PDA/UVvis detector, and a LCQ Advantage ion trap ESI mass spectrometer (ThermoFinnigan, San Jose, CA, USA) equipped with Xcalibur 3.1 software. Qualitative analysis was performed on a C-18 column (Luna, 4.6 × 150 mm, 5 µm, Phenomenex, Bologna, Italy) using a mixture of methanol (solvent A) and formic acid in water 0.1% *v/v* (solvent B) as eluent. A linear gradient of increasing 5 to 55% A was developed within 50 min at a flow rate of 0.8 mL/min, with a splitting system of 2:8 to MS detector (160 μL/min) and PDA detector (640 μL/min), respectively. PDA/UV spectra were recorded in a range of 200–600 nm, using 254, 280, and 325 nm as preferential channels, while ESI-MS experiments were achieved in negative ion mode (scan range of *m/z* 150-2000). Ionization parameters were used as previously reported [30].

### 2.4. Antioxidants Determination

The total antioxidant potential of cocoa by-product extracts and CE freeze-dried samples was determined by a ferric reducing antioxidant power (FRAP) assay, as previously reported [31]. The FRAP value of the samples, expressed as mmol of Fe^2+^ per 100 g FW, was determined from a standards curve built up using ferrous sulfate.

### 2.5. Total Polyphenolic Content

The total polyphenols content (TPC) of cocoa by-product extract and CE was determined by the Folin–Ciocalteau method [32]. The results were expressed as gallic acid equivalent (GAE) on a dry weight basis following a previously reported procedure [33].

### 2.6. HUVEC Isolation and Culture

HUVECs were collected from umbilical cords obtained from healthy donors women post-labor, treated anonymously. The age of the donors ranged from 24 to 43 years. Ethical approval (authorization number 2943), was granted by the local Ethics Committee (Full name: Comitato Etico per la Sperimentazione Clinica Area Vasta Nord Ovest c/o Azienda Ospedaliero-Universitaria Pisana (AOUP), Pisa”). HUVECs were collected following the procedure described by Jeffe et al. [34]. Briefly, HUVECs after isolation and centrifugation were plated on gelatin pre-coated flasks and incubated for 24 h at 37 °C, using 5% CO_2_ in a complete growth medium made with 10% FBS. After 24 h, the growth medium was replaced to remove the excess of red blood cells.

### 2.7. Cell Treatment

HUVECs between passage P2–P4 were treated for 2 h and 24 h with increasing polyphenol concentrations of cocoa husk Costa Rica extract (CHE) and Crognola Capannile cherry extract (CE) (5, 10, 25 or 50 μg GAE/mL), in growth medium with 5% FBS. Cells in medium only were used as a positive control. Then, cells were washed with PBS and treated with 50 µM of commercial H_2_O_2_ for 1 h to induce oxidative stress [33]. At the end of each treatment, cells were analyzed for viability and ROS production.

### 2.8. Cell Viability

At the end of each treatment, HUVECs were incubated with tetrazolium salt (10 µL/well) for 3 h at 37 °C, in 5% CO_2_ and the formazan dye formed was quantified at 450 nm with a multiplate reader (Thermo Scientific Multiskan FC Microplate Photometer), correlating the with the number of active cells. The viability was expressed as the percentage of viable cells.

### 2.9. ROS Production

ROS production was evaluated using CM − -H2DCFDA, a fluorescent probe. HUVECs during the last 15 min of treatment with CHE, CE or H_2_O_2_, were incubated in the dark at room temperature with CM − -H2DCFDA (10 µL/well) dissolved in PBS. ROS production was detected measuring the increase in fluorescence over time at excitation of 488 nm and emission of 510 nm using a microplate reader (Thermo Scientific Fluoroskan Ascent Microplate Fluorometer). ROS production was expressed as a percentage of ROS accumulation.

### 2.10. Permeation Study of CHE and CE

For the ex-vivo permeation studies, we used the intestinal mucosa excised from non-fasting male Wistar rats (weight 250–300 g), using the procedure reported by Fabiano et al. [35]. The ex vivo experiments on excised rat intestine were carried out with the aim of assessing the permeability of the intestinal epithelium to the antioxidants present in the different extracts. The isolated rat intestine was chosen from among the known intestinal models because its tight junctions are similar in number and tightness to those of the human intestine [36]. The formulation tested were 1 mL of CE solution (15.5 µg/mL GAE concentration [33], or CHE (280 µg/mL GAE concentration). At 30 min intervals of a total of 240 min, the apical to basolateral transport of CHE or CE was investigated, analyzing the receiving phase (50 µL) by the Folin–Ciocalteau reagent for TPC.

### 2.11. Cocoa Extract and CE Stability Studies

The stability of CHE and CE was evaluated according to the procedure reported by Beconcini et al. [33]. At 30 min intervals of a total of 240 min, 50 µL of CHE or CE volume was withdrawn and analyzed by the Folin–Ciocalteau reagent for TPC.

### 2.12. Statistical Analysis

The GraphPad Prism Software vs. 7.0 (GraphPad Software Inc., La Jolla, CA, USA) was used for the statistical analysis of data. All results were presented as means ± standard deviation (SD) of at least three independent experiments. The significant difference (*p*-value < 0.05) between groups of values was evaluated by a one-way ANOVA or Turkey’s or Bonferroni’s multiple comparisons.

## 3. Results

### 3.1. Phenolic Profile of Cocoa Extracts

**Figure 1:**
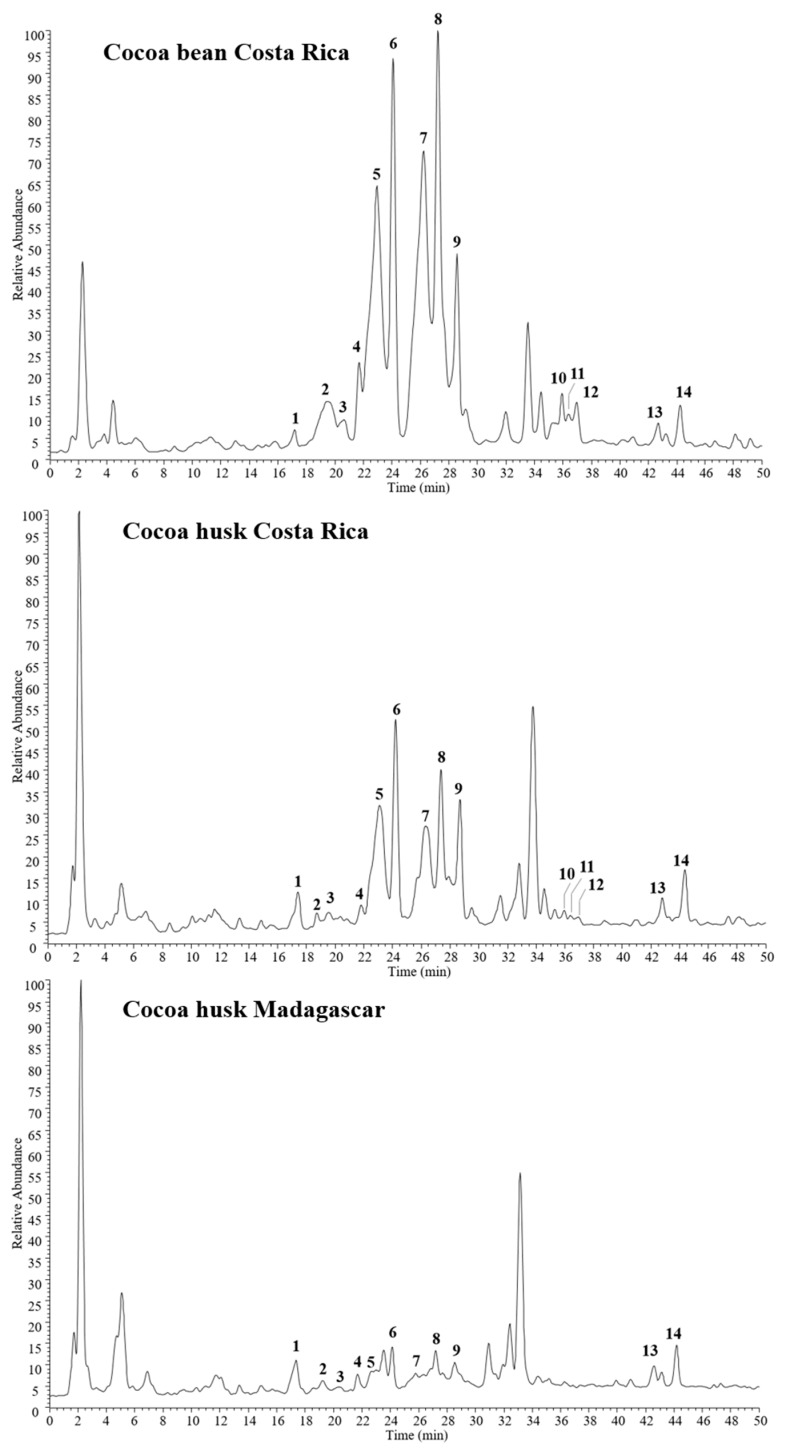
HPLC-electrospray ionization (ESI)-MS/MS profiles of phenols detected in cocoa husk and bean extracts in negative ion mode. Peak data are listed in Table 1.

The phenolic composition of cocoa bean and husk extracts was characterized by HPLC-PDA/UVvis-ESI-MS/MS experiments. Cocoa Costa Rica extracts (bean and husk) showed similar chemical profiles (Figure 1), with 14 major phenol compounds identified: one caffeoyl derivative, caffeoyl aspartate (**1**); one flavan-3-ol, catechin/epicatechin (**9**); three procyanidin B isomers (**5**, **6**, and **10**) together with seven procianydin C isomers (**2**, **3**, **4**, **7**, **8**, **11**, and **12**); two flavonol glycosides, quercetin 3-O-glucoside (**13**), and quercetin 3-O-arabinoside (**14**).

In contrast, the Madagascar cocoa husk extract showed a less rich phenolic content, mainly with regard to procyanidins. Indeed, compounds **5**-**9**, which were the most representative constituents in cocoa husk Costa Rica extract, were significantly reduced in Madagascar cocoa husk, while compounds **10**, **11**, and **12** were not detected at all. These findings are in agreement with previous studies, reporting a high different phenol content in cocoa products with different geographic origins, i.e., Costa Rica and Madagascar [24].

All compounds were tentatively identified by comparison of their elution order, UV data, and both full and fragmentation mass spectra (Table 1) with data reported in the literature [13,37,38]. Compounds **2–4**, **7**, **8**, **11**, and **12** showed the same molecular deprotonated ion [M − -H]^−^ at *m/z* 865 and two strong UV absorptions at 242–258 and 277–280 nm, suggesting a trimeric B-type procyanidin structure for all isomers. This finding was confirmed by fragmentation mass spectra, all showing the same diagnostic product ions at *m/z* 739, 713, 695, 451, 407, and 287, together with a base peak ion at *m/z* 577, represented by the dimeric form [30].

Since all the spectra are superimposable, it is not possible to distinguish between the isomeric forms [39], but all compounds can be assigned as procyanidin C isomers, previously found in cocoa extract [37]. Likewise, full MS of peaks **5**, **6**, and **10** were characterized by type-B procyanidin dimers, as deduced by the deprotonated molecule [M − -H]^−^ at *m/z* 577 and product ions at *m/z* 451, 425, 407, and 289. Also, in this case, the exact structure cannot be assigned only on the basis of spectral data. However, it can be assumed all three molecules to be isomers of procyanidin B, previously reported in cocoa extract [37]. In addition to these oligomers, also their monomer was detected (compound **9**, [M − -H]^−^ at *m/z* 289), corresponding to catechin or epicatechin, as deduced by diagnostic product ions at *m/z* 245 and 205 [30].

Along with flavan-3-ols, also two flavonol glycosides were identified in all three analyzed cocoa extracts, as evidenced by UV absorptions at 267–268 and 354–355 nm. Compounds **13** ([M − -H]^−^ at *m/z* 463) and **14** ([M − -H]^−^ at *m/z* 433) were quercetin derivatives showing the loss of a hexose ([M − -H-162]^−^ at *m/z* 301) and a pentose units ([M − -H-132]^−^ at *m/z* 301), respectively. According to data reported in previous work [40], **13** and **14** were identified as quercetin 3-*O*-glucoside and quercetin 3-*O*-arabinoside, respectively.

Finally, a caffeoyl derivative (compound **1**, [M − -H]^−^ at *m/z* 293) was detected in all cocoa samples, showing the loss of an aspartate residue ([M − -H-115]^−^ at *m/z* 301) in the ESI-MS/MS experiments. Thus, compound **1** was identified as *N*-caffeoyl aspartate, previously reported in cocoa source [37]. Some other peaks remained unidentified; however, based on UV data, they were not attributed to phenol derivatives.

### 3.2. Cherry and Cocoa By-Product Extracts Characterization

FRAP and Folin–Ciocalteu methods on CE reported that Crognola had the highest antioxidant content and TPC (402.5 ± 8.4 mg GAE/100 g FW) among the six varieties of Prunus avium L. studied. Flavonoid molecules of quercetin and catechins were 59.32 ± 3.2 µg/g FW and 292.76 ± 1.9 µg/g FW, respectively. HPLC analysis also showed the presence of anthocyanins, represented mainly by cyanidin-3-glucoside (227.37 ± 1.2 µg/g FW).

In Table 2, the antioxidant content and the TPC of the two extracts under study are reported. CE and CHE antioxidant content was not significantly different (*p* > 0.05), whereas CHE had a higher TPC than CE (*p* < 0.05).

### 3.3. Dose- and Time-Dependent Effect of CHE and CE on HUVECs Viability

**Figure 2:**
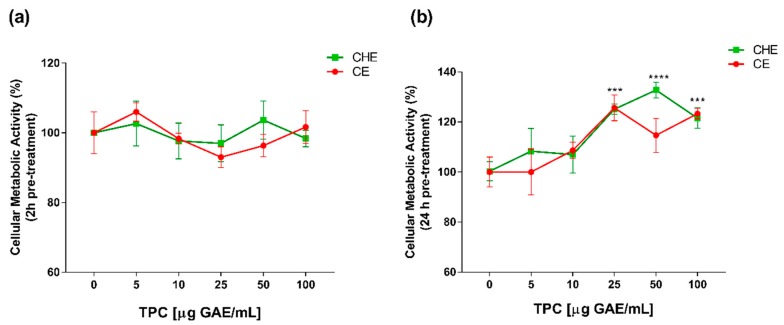
Dose- and time-dependent cell metabolic activity. Human Umbilical Vein Endothelial Cells (HUVECs) were cultured for 2 h (**a**) and 24 h (**b**) in the presence of increasing concentrations of total polyphenol content (TPC) from CE or CHE (5, 10, 25, 50, and 100 µg GAE/mL). Cell metabolic activity was determined by WST-1 colorimetric assay and expressed as metabolic activity percentage compared to control (untreated cells). Graphical data are represented as mean ± SD of three separate experiments run in triplicate. (*** *p* < 0.005, **** *p* < 0.0001 vs. control).

Polyphenolic concentrations of 5, 10, 25, 50 and 100 µg GAE/mL, were chosen for viability studies. Cell viability was evaluated by WST-1 colorimetric assay. Both CHE and CE polyphenols were non-cytotoxic, both after 2 h and 24 h of treatment (Figure 2a,b). After 24 h of treatment, there was an increase in cell viability at high concentrations of CHE (25 to 100 µg GAE/mL TPC) compared to control (Figure 2b). This enhancement of cell viability might be due to the incubation over time addicted to a higher concentration of polyphenols, as previously demonstrated in apple juice study on HUVECs [20].

### 3.4. Protective Effect from Oxidative Stress

To evaluate the antioxidant activity of CHE and CE, HUVECs were pre-treated for 2 h and 24 h with increasing polyphenols concentrations (ranging from 5–100 µg GAE/mL of TPC), and after that, an oxidative stress insult was applied by treating the cells with 50 µM H_2_O_2_ for 1 h.

**Figure 3:**
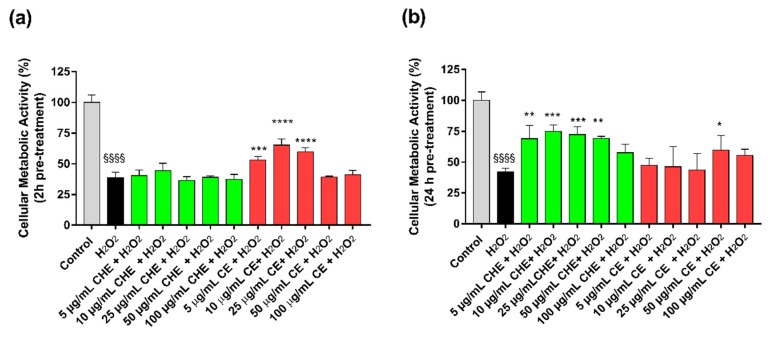
Protective effects from H_2_O_2_-induced oxidative stress. HUVECs viability after 2 h (**a**) or 24 h (**b**) of pre-treatment with CE or CHE and treatment with 50 µM H_2_O_2_ for 1 h. Data are expressed as the % of viable cells compared to 100% of control (untreated cells). (*p < 0.05, ** *p* < 0.005, *** *p* < 0.0005, **** *p* < 0.0001 vs. H_2_O_2_; ^§§§§^ *p* < 0.0001 vs. control).

The results reported that treatment of HUVECs with H_2_O_2_ significantly reduced viable cell number compared to control (Figure 3). Viability after H_2_O_2_-induced oxidative stress was increased by a pre-treatment with CE, both after 2 h or 24 h (Figure 3b). In particular, after 24 h, only 50 µg GAE/mL TPC of CE mL had the most significant protective effect. For CHE polyphenols, only the 24 h pre-treatment significantly protected cells from oxidative stress in a concentration range of 5 µg GAE/mL TPC to 50 µg GAE/mL TPC.

### 3.5. Antioxidant Activity

**Figure 4:**
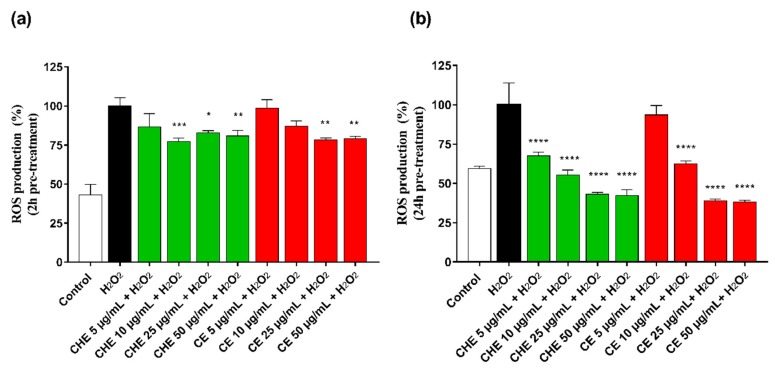
Rective Oxygen Species (ROS) production by HUVECs was evaluated after 2 h (**a**) and 24 h (**b**) of incubation with different concentrations of CHE and CE (i.e., 5, 10, 25, 50, and 100 µg/mL of TPC) and 100 µM H_2_O_2_ for 1 h. Data are expressed as ROS production% by treated cells and are representative of three separate experiments run in triplicate (* *p* < 0.05, ** *p* < 0.005, *** *p* < 0.0005, **** *p* < 0.0001 vs. H_2_O_2_).

ROS accumulation in HUVECs was evaluated after 2 h and 24 h pre-treatment of CHE or CE at different concentrations (ranging 5–50 µg GAE/mL of TPC). Treatment of HUVECs with H_2_O_2_ significantly increased intracellular ROS production. As shown in Figure 4a, after 2 h pre-incubation, CHE showed to significantly reduce ROS production already at low concentration (10 µg GAE/mL TPC) compared with control, while a significantly protective effect by CE polyphenols was observed at a concentration above 25 µg GAE/mL (** *p* < 0.005 vs. control).

CHE and CE seemed to increase their antioxidant power over incubation time, as observed in Figure 4b, and directly correlated to their polyphenolic content, indicating a time- and dose-dependent effect on cell metabolic activity.

### 3.6. Permeation Study of CHE and CE

For each permeation run, the value of the apparent permeability coefficient, P’_app_, for permeant across the excised rat intestinal mucosa was calculated from the following equation

**Figure 5:**
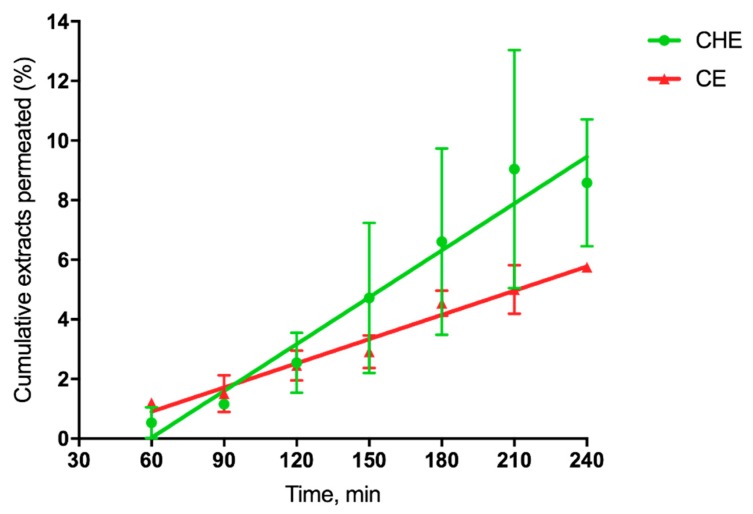
Cumulative CHE and CE permeated (%) across excised rat intestine.

P’_app_ = dM/dt × 1/(AC_0_) (1)

where dM/dt 1/A, the permeation flux, is the slope of the linear portion of the cumulative amount permeated per unit surface area vs. time plot, and C_0_ is the CHE or CE concentration (280 µg/mL or 15.5 µg/mL, respectively) introduced at the donor phase. For each plot, the quality of the linear regression, shown in Figure 5, as evidenced by r^2^ values being >0.9.

Such linearity allowed the application of Equation (1), as reported by [41]. The single P’_app_ values were averaged to calculate the mean apparent permeability P_app_ (n = 3). Since the extract concentration was different for CHE and CE, we calculated the cumulative percentage permeated at each time, useful for comparative purposes. The relevant data reported in Table 3 shows that for cocoa extract, the apparent permeability was 2-fold higher than that corresponding to CE, as the flux was 36-fold higher than that of CE.

### 3.7. CHE and CE Stability Studies

**Figure 6:**
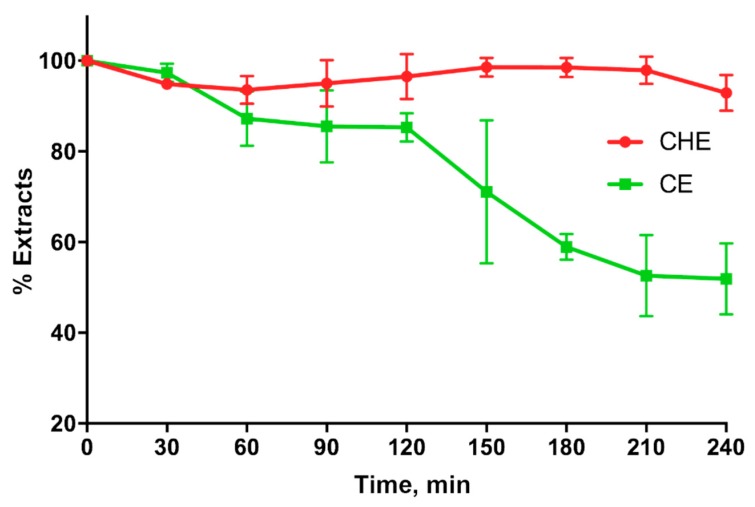
Cocoa or CE stability in simulated gastric fluid.

CHE and CE stability in simulated gastric fluids (SGFs) shown in Figure 6, indicates that CHE is more stable than CE in the stomach for at least 4 h. In fact, the CHE polyphenols percentage in the SGF was around 100% for the entire duration of the experiment, whereas that corresponding to CE decreased around 50% just after 3 h.

## 4. Discussion

Natural products are increasingly used in scientific research for their potential effect on human health. Many studies show their beneficial effects in clinical and in vitro studies due to the presence of antioxidant molecules, mainly polyphenols. Polyphenols have shown their effects against chronic diseases, including CVD. For the treatment of CVD, prevention plays an important role. The introduction of nutraceuticals in the diet could represent the first defense mechanism of the body from oxidative stress.

In the present study, we compare the antioxidant effects of the molecules contained in an ancient variety of Tuscan cherries (Crognola Capannile) and cocoa by-product extracts (bean and husk).

Numerous studies reported that sweet cherries had high and variable concentrations of antioxidants and TPC in different varieties. A recent study by Berni et al., found the same differences in Tuscan cherries varieties [22]. The analysis confirmed the presence of a high content of antioxidant and phenolic compounds, particularly in the variety Crognola Capannile for CE and Costa Rica for cocoa by-products extract, mainly husks. Specifically, the characterization of the phenolic composition of cocoa bean and husk extracts demonstrated a high concentration, mainly with regard to procyanidins. Above all, compounds **5**–**9** were the most representative constituents in cocoa husk Costa Rica extract. According to previous studies, cocoa extracts were found to be rich in flavonoids, in particular, dimeric and trimeric procyanidins, among which, the B-type (characterized by a C4–C8 or C4–C6 bond between monomers catechin/epicatechin) were the most representative [37].

Several studies have tested natural products-derived polyphenols on HUVECs [19,20,21] for in vitro experiments related to vascular dysfunction. In this study, we have investigated the in vitro properties of Crognola CE, obtained from fresh fruits, and selected from the six Tuscan varieties the highest TPC and molecular content (as previously shown by Berni et al. [22]), and Costa Rica cocoa by-product extract (in particular, husk product (CHE)), for their rich phenolic content [25].

Vascular oxidative stress contributes to mechanisms of vascular dysfunction and has been implicated in playing an important role in a number of cardiovascular pathologies [42]. There are also many in vitro studies about the antioxidant properties of *Prunus avium* L. and cocoa by-products for the prevention of chronic diseases [12,16,43,44]. The presence of hydroxycinnamic acids, flavonoids and anthocyanins made CE interesting for in vitro experiments related to oxidative stress. Matias et al. [16] demonstrated that a 2 h pre-treatment with cherry extracts effectively alleviated oxidative stress caused by H_2_O_2_-induced injury in neuronal cells.

In a recent study, Rebollo–Hernanz et al. [44] demonstrated that cocoa by-product extracts effectively reduced inflammatory markers in macrophages and adipocytes and the production of reactive oxygen species. Moreover, extracts from cocoa by-products also modulated the phosphorylation of the insulin receptor signaling pathway and stimulated GLUT-4 translocation, increasing glucose uptake [44].

Our results show that lower concentrations of CE protected from oxidative stress in a shorter treatment time (2 h, Figure 3a), in agreement with other studies [16], whereas a higher concentration of polyphenols was required in long-term treatment (24 h, Figure 3b). These results demonstrate the indirect antioxidant potential of CHE, possibly acting through the augmentation of cellular antioxidant capacity by enhancing specific genes encoding antioxidant proteins [45].

Polyphenols showed an anti-radical activity supported by different studies [12,46,47]. In this study, the antioxidant activity of CHE and CE polyphenolic molecules has been verified through the evaluation of ROS production, both with and without H_2_O_2_-stress induction on HUVECs.

The obtained results suggest that polyphenols in sweet CE and CHE are able to inhibit ROS, protecting cells from oxidative stress. In particular, CHE showed a time- and dose-dependent effect on cell metabolic activity, probably due to an indirect antioxidant effect [45]. Specifically, CHE showed an antioxidant effect at low concentrations.

Finally, in the present study, we evaluated the ability of natural extracts to cross the excised intestinal wall. The results indicate that CHE is more able to permeate through the excised intestinal wall compared to CE, probably because the antioxidants contained in CHE are more stable than those in CE when they come into contact with the rat intestinal tissue. This hypothesis is in agreement with the results shown in Figure 6, indicating that CHE is more stable than CE in the gastric environment.

## 5. Conclusions

The two extracts studied in this work, CHE and CE, have both been shown to have antioxidant activity on HUVECs, i.e., on the cells of the endothelium of blood vessels, thus proving to be potential compounds for the prevention of CVD. Between the two extracts, CHE showed better performance on HUVECs, and greater permeability across the rat intestine than CE, perhaps due to its greater stability in the physiological environment. The results obtained in this work encourage us to continue the studies on cocoa husks extracts, which represent a waste product of cocoa processing and a cost for their disposal. Thanks to their beneficial properties, cocoa by-products might become a nutraceutical research topic for possible medical applications in CVD, as well as a potential ingredient for the food and pharmaceutical industries.

## Figures and Tables

**Table 1:** ESI-MS/MS, UV, and chromatographic data (retention time, *t*_R_) of compounds **1**-**14** detected in the cocoa bean and husk extracts Costa Rica and cocoa husk Madagascar.

Peak ^a^	Compound	*t*_R_ (min)	M	[M+HCOO]^−^	[M − H]^−^	ESI- MS/MS (Product Ions) (*m/z*) ^b^	UV (λ_max_)
	Phenols						
**1**	*N*-caffeoyl aspartate	17.8	295		294	276, **179**, 132	252, 277, 305
**2**	procyanidin C (trimer I)	19.4	866		865	847, 739, 713, 695, **577**, 451, 407, 287	258, 277
**3**	procyanidin C (trimer II)	20.7	866		865	847, 739, 713, **695**, 577, 451, 407, 287	252, 279
**4**	procyanidin C (trimer III)	21.7	866		865	847, 739, 713, **695**, 577, 451, 407, 287	248, 280
**5**	procyanidin B (dimer I)	23.0	578		577	451, **425**, 407, 289	243, 279
**6**	procyanidin B (dimer II)	24.1	578		577	451, **425**, 407, 289	243, 279
**7**	procyanidin C (trimer IV)	26.3	866		865	847, 739, 713, **695**, 577, 451, 407, 287	244, 280
**8**	procyanidin C (trimer V)	27.2	866		865	847, 739, 713, **695**, 577, 451, 407, 287	242, 279
**9**	catechin/epicatechin	28.6	290	335	289	271, **245**, 205, 179	240, 279
**10**	procyanidin B (dimer III)	36.0	578		577	451, **425**, 407, 289	277
**11**	procyanidin C (trimer VI)	36.4	866		865	739, 713, **695**, 577, 451, 407, 287	277
12	procyanidin C (trimer VII)	37.0	866		865	739, 713, **695**, 577, 451, 407, 287	278
**13**	quercetin 3-*O*-glucoside	42.7	464		463	**301**, 179	268, 355
**14**	quercetin 3-*O*-arabinoside	44.2	434		433	**301**, 179	267, 354

^a^ Peak numbers correspond with those of Figure 1. ^b^ Ions were generated by fragmentation of molecular deprotonated ions in the ESI-MS/MS experiments, and the base peaks are showed in bold.

**Table 2:** Cherry Extract (CE) and Cocoa Husk Extract (CHE) characterization. ^a^ Determined by FRAP. ^b^ Determined by Folin–Ciocalteau. * Significantly different from each other (*p* < 0.05). TPC = total polyphenols content.

Extract	Antioxidant Content ^a^ (mmol Fe^2+^/100 g FW)	TPC ^b^ (mg GAE/100g FW)
CE	2.19 ± 0.09	402.5 ± 8.4
CHE	2.50 ± 0.01	7105 ± 96.9 *

**Table 3:** Data on CE or cocoa extract permeation across the excised rat intestine. ^a^ Apparent permeability. ^b^ Cumulative extract permeated (%) over the whole experiment time (4 h). * Significantly different from each other (*p* < 0.05).

Extract	Flux 10^2^ (µg cm^−2^min^−1^)	P_app_^a^ 10^4^ (cm min^−1^)	T_4h_^b^ (%)
**CE**	0.41 ± 0.03	2.64 ± 0.02	5.75 ± 0.07
**CHE**	14.50 ± 1.33 *	5.18 ± 0.47 *	8.58 ± 2.13
